# Spider phobics more easily see a spider in morphed schematic pictures

**DOI:** 10.1186/1744-9081-3-59

**Published:** 2007-11-19

**Authors:** Iris-Tatjana Kolassa, Arlette Buchmann, Romy Lauche, Stephan Kolassa, Ivailo Partchev, Wolfgang HR Miltner, Frauke Musial

**Affiliations:** 1 Institute of Psychology, Biological & Clinical Psychology, Friedrich Schiller University Jena, Am Steiger 3, 07743 Jena, Germany.; 2 Clinical & Neuropsychology, University of Konstanz, P.O. Box 5560, 78457 Konstanz, Germany.; 3 Operations Research, Institute of Applied Mathematics, Friedrich Schiller University Jena, Ernst-Abbe-Platz 2, 07743 Jena, Germany.; 4 Institute of Psychology, Methodology & Evaluation Research, Friedrich Schiller University Jena, Am Steiger 3, 07743 Jena, Germany.

## Abstract

**Background:**

Individuals with social phobia are more likely to misinterpret ambiguous social situations as more threatening, i.e. they show an interpretive bias. This study investigated whether such a bias also exists in specific phobia.

**Methods:**

Individuals with spider phobia or social phobia, spider aficionados and non-phobic controls saw morphed stimuli that gradually transformed from a schematic picture of a flower into a schematic picture of a spider by shifting the outlines of the petals until they turned into spider legs. Participants' task was to decide whether each stimulus was more similar to a spider, a flower or to neither object while EEG was recorded.

**Results:**

An interpretive bias was found in spider phobia on a behavioral level: with the first opening of the petals of the flower anchor, spider phobics rated the stimuli as more unpleasant and arousing than the control groups and showed an elevated latent trait to classify a stimulus as a spider and a response-time advantage for spider-like stimuli. No cortical correlates on the level of ERPs of this interpretive bias could be identified. However, consistent with previous studies, social and spider phobic persons exhibited generally enhanced visual P1 amplitudes indicative of hypervigilance in phobia.

**Conclusion:**

Results suggest an interpretive bias and generalization of phobia-specific responses in specific phobia. Similar effects have been observed in other anxiety disorders, such as social phobia and posttraumatic stress disorder.

## Background

Cognitive biases have been assumed to play an important role in the development and maintenance of anxiety disorders. Biases in anxiety disorders have been broadly categorized in empirical research as biases affecting the three general stages of information processing (1) attention and the encoding of information; (2) elaboration and interpretation; and (3) storage and retrieval from memory [1,2].

An example for an interpretive/judgmental bias is the negative interpretation bias found in particular in social phobics. Several studies showed that individuals with social phobia are more likely to misinterpret (ambiguous) social situations as more threatening and to draw more negative inferences from social stimuli than controls [3-5]. In spider phobia, Becker & Rinck [6] found a generalized interpretive bias by presenting pictures of spiders, beetles or butterflies interspersed with neutral pictures for 14 ms each. Spider phobic participants were more likely to report having seen a spider or a beetle, which was interpreted as applying a more liberal criterion both to highly negative spiders and to slightly negative beetles.

The concept of stimulus generalization, first introduced by Pavlov [7], is closely related to interpretive biases. Stimulus generalization refers to the fact that conditioning of a particular stimulus will result in generalization of this conditioning to other, similar stimuli. This generalization leads to similar yet weaker responses to new stimuli compared to the originally conditioned stimulus [e.g., [8]].

### Processing of fear-relevant stimuli in specific phobia

Several PET, functional MRI and ERP studies have investigated the processing of fear-relevant stimuli in phobic patients (e.g. [9-15]). Fredrikson et al. [9] were among the first to report elevated regional cerebral blood flow (rCBF) in the visual associative cortex of snake phobics who viewed phobic, as compared to neutral and aversive stimuli. The elevated rCBF in the visual cortex of phobics in response to their feared object [9,10] is in line with studies reporting more extensive activation of the visual cortex when viewing highly emotional (arousing) stimuli [e.g., [16,17]].

Similarly, ERP studies revealed enlarged late positive potentials in spider phobic individuals in response to feared objects [13-15]. These results are in accordance with the larger parietal cortical positivities observed in response to highly emotional (arousing) stimuli in non-phobic individuals (e.g., [18,19]). Whereas the influence of emotional valence/arousal on late ERP components is well-documented, early ERP components have not been fully investigated. Miltner et al. [15] observed no phobia-specific effect on early ERP components (N1, P2, N2) when spider or snake phobic individuals were processing pictures of feared objects. However, in a study investigating the processing of schematic spider and flower stimuli consisting of the same visual elements, Kolassa et al. [14] found generally enhanced P100 amplitudes in individuals with spider phobia and individuals with social phobia, as compared to non-phobic controls. These observations were interpreted as evidence for an increased (cortical) hypervigilance for incoming stimuli in phobic patients in general. Furthermore, all groups, whether spider phobic or not, showed faster identification of and larger N170 amplitudes in response to schematic spider versus flower pictures, which may reflect a general advantage in the processing of fear-relevant features.

### Fear-relevant features and Gestalt properties

Öhman [20] postulated the existence of specific feature detectors that are preferentially sensitive to elementary threat features which were significant for survival during evolution. If such a threat feature is detected, the stimulus automatically and preattentively activates the arousal system and becomes tagged for preferential evaluation by a succeeding significance evaluation system [20]. However, as Öhman et al. [[21], p. 475] admit, "such elementary threat features [...] still remain to be specified." As facial expressions signaling social threat should presumably have been of evolutionary significance, in recent years facial features that convey threat have been extensively investigated [22-25]. However, which properties make a spider fear-relevant is still unknown. Is it the shape of the body of a spider, its protruding legs, the angle in which the legs are positioned in relation to each other and to the body? Or is it the movement of a spider or a snake that is detected by these feature detectors? Or do the feature detectors respond to still other details of the feared stimulus?

**Figure 1:**
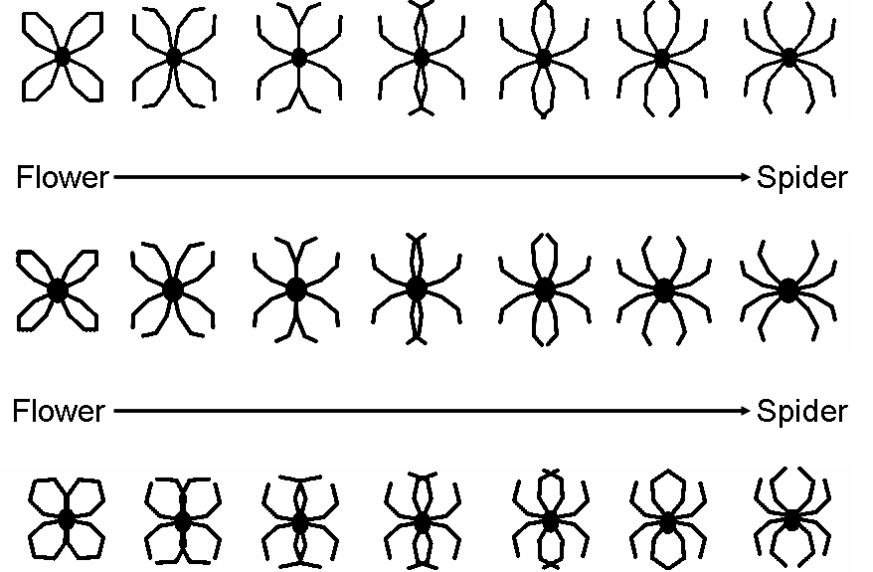
**Stimuli**. Three series of schematic flower/spider stimuli: starting from the picture of a schematic flower, the stimuli gradually turned into a spider by shifting the outlines of the petals until they turned into spider legs.

The present study attempts to partly fill this gap by investigating the role of the Gestalt of a spider as one of the fear-inducing properties that might induce fear in spider phobic subjects. "Gestalt" refers to the perception of a whole object as a result of the relation of each of its parts to each other [26], in contrast to single features such as color or brightness. For this purpose, three series of schematic flower/spider pictures were designed: flower anchors differed in size of the interior of the flower and the angularity of the outlines of the petals, while spider anchors differed in body size and angularity of spider legs (see Figure 1). Each series contained seven pictures that stepwise morphed a schematic flower into a schematic spider by gradually shifting the angles of the outlines of the petals into open patterns that corresponded to spider legs (see Figure 1).

Four groups of subjects participated in this study: individuals with spider phobia, spider aficionados, individuals with social phobia, and non-phobic controls. Both social phobic and spider phobic individuals have an anxiety disorder, but they do not share the specific phobia-relevant object or situation. On the other hand, spider aficionados share with spider phobic persons the subjective significance of spiders without interpreting spiders as objects of threat. Therefore, the present set of subjects allows to separate the effects of relevance (threat meaning) and anxiety.

All subjects rated each of the stimuli according to their valence and arousal. In the actual paradigm, subjects repeatedly classified the stimuli into one of the three categories "flower", "spider" and "neither/nor" while ERPs were measured. Dependent variables were valence and arousal ratings, reaction times, classification frequencies, and event-related potentials in response to each of the different stimuli.

### Aims and hypotheses

We expected an interpretive bias, particularly for ambiguous ("in-between") stimuli, in spider phobic individuals. This interpretive bias should manifest in all dependent variables, i.e., stimuli with a Gestalt between flower and spider should more likely be rated as more unpleasant and more arousing and be classified earlier as spiders by spider phobics than by controls. Furthermore, spider phobic subjects should show faster responses to pictures which they classify as spider-like. Additionally, spider phobics should show larger N170 and parietal late positive potentials (LPPs) amplitudes to ambiguous stimuli than non-fearful subjects and spider aficionados. In addition, we expected larger P1 magnitudes in phobic than in non-phobic participants.

## Methods

### Participants

Fifty-nine subjects (age range 18–34 yrs, *M *= 23.5 years, *SD *= 3.8) participated in the study: 16 individuals with spider phobia (8 male, 8 female), 15 spider aficionados (6 male, 9 female), 13 individuals with social phobia (6 male, 7 female), and 15 non-phobic controls (9 male, 6 female). Fifty-seven subjects were right-handed and 2 were left-handed, as measured by the Edinburgh handedness questionnaire [27]. Subjects were recruited by newspaper advertisement and within the university student population.

Prior to the experiment proper, participants were screened with the Structured Clinical Interview for DSM-IV [SCID-I, [28]]. Spider phobic and social phobic individuals were included in the study if they fulfilled DSM-IV criteria [29] of spider phobia or social phobia, respectively, without any other current or previous disorders according to DSM-IV. Candidate spider aficionados were recruited by specific advertisements for "individuals who like spiders". Only applicants who habitually handled spiders or owned one or more spiders as pets were selected for participation. They were administered a behavioral test in which they were confronted with a garden spider (*Araneus diadematus*) that they had to be able to pick up unhesitatingly and without any sign of fear or disgust and to keep in their hands for a couple of minutes without fear responses. Controls and spider aficionados were accepted for participation if they had no current or past mental disorders according to DSM-IV. All study participants were free of any psychotropic medication.

Prior to the experiment, all participants completed German versions of the Spider Questionnaire [SPQ, [30]], the Social Phobia and Anxiety Inventory [SPAI, [31]], the Beck Depression Inventory [BDI, [32]] and the Trait Anxiety Questionnaire of the State-Trait Anxiety Inventory [STAI, [33]]. Spider aficionados had lower SPQ values than controls and social phobic participants, Mann-Whitney *U *= 123.00, *p *= .02. Social phobic individuals, on average, showed higher BDI scores than the other groups. However, all social phobics had BDI scores ≤ 15 and were thus in a clinically non-significant range. See Table 1 for questionnaire values.

**Table 1:** Mean questionnaire values (M) and standard deviations (SD) for each group

	Control Group		Spider Phobics		Spider Aficionados		Social Phobics		
		
Questionnaire	M	SD	*M*	*SD*	*M*	*SD*	*M*	*SD*	Kruskal-Wallis Test
SPQ	2.13	1.73	20.31	2.60	.93	1.10	2.23	1.96	*χ*^2^(df = 3) = 37.76, *p *< .0001
SPAI	37.06	17.69	45.88	13.54	46.64	18.33	128.19	17.46	*χ*^2^(df = 3) = 31.66, *p *< .0001
BDI	3.60	3.44	4.81	4.13	4.73	3.71	8.62	5.17	*χ*^2^(df = 3) = 7.293, *p *< .05

The German scores of the Social Phobia and Anxiety Inventory (SPAI) were transformed into the original scores (Turner et al., 1989). STAI-T, State-Trait Anxiety Inventory – Trait Version; BDI, Beck Depression Inventory; SPQ, Spider Questionnaire.

In the SPQ, spider phobic individuals differed from social phobic, spider aficionado and control groups, all *p *< .0001, and spider aficionados differed from controls, *p *= .04. In the SPAI, social phobic participants differed from all other groups, all *p *< .0001. In the BDI, social phobic individuals differed from controls, *p *= .01, spider aficionados, *p *= .03, and spider phobic participants, *p *= .05.

All participants provided informed consent, and the procedures were approved by the ethics committee of the Friedrich Schiller University Jena. Subjects were paid 6 € per hour for participation. Additionally, social phobic participants were offered a 10-session group training for social skills [34] and spider phobic individuals could participate in a one-day spider phobia treatment [35].

### Valence and arousal ratings of stimuli

Prior to the main experiment, participants rated all stimuli as to their affective valence and physiological arousal using the Self-Assessment Manikin scale [36,37]. The order of stimuli was randomized across participants.

### Paradigm

Participants' task was to decide whether the stimuli were more similar to a "spider", a "flower", or "neither/nor" by pressing corresponding buttons on the arm-rests. There were two buttons on each armrest. Of these 4 buttons, 3 were used (Spider, Flower, Neither/Nor). Subjects pressed these buttons with the index and middle fingers of their right and left hand. The keys which had to be pressed to classify the stimuli were randomized across subjects. A sheet of paper showing the correspondence between keys and answer categories lay in front of them; however, subjects were instructed to look at this sheet only if absolutely necessary.

Before the experiment started, participants performed two training tasks of 9 trials each which they could repeat as often as necessary. Before each trial, the key sequence for the buttons mounted on the armrests was shown for 4 s on the screen. Then one of the stimuli was presented on the screen for 3 s, and subjects indicated their classification by pressing the appropriate button. Participants were instructed to respond as quickly as possible. After each classification the subject confirmed, again by pressing a button, whether the response had been correct or incorrect. Although there were no predefined right or wrong answers, subjects could have mistakenly pressed the wrong button. This procedure allowed subjects to identify unintended false responses so that trials with false responses could be removed from analyses. During the second training trials no key sequence was shown and the confirmation screen was simplified to "Correct?".

In, the experiment proper, each picture of the three flower/spider series was presented 14 times. To avoid fatigue, the paradigm was split into two blocks with a short break between them. Stimuli were presented in a pseudo-random order in which no picture appeared twice in a row. Each stimulus was presented for 3 s, during which subjects gave their answer by pressing the appropriate key. In the inter-stimulus interval of 2 s ± 400 ms (1600 ms plus an exponential distribution with mean 400 ms, truncated at 800 ms, as generated by ERTS), the question "Correct?" appeared on the screen, and subjects indicated by pressing a button whether the answer given had been right or wrong.

### Assessment and analysis of EEG

During the testing session, subjects sat in a comfortable chair in a sound-attenuated room. Stimuli were presented on a 20 inch Sony monitor (resolution 800 × 600) placed 1.1 m in front of the subject's eyes, using ERTS (Experimental Runtime System [38]). EEG was recorded with 62 Ag/AgCl-electrodes mounted in an Easy-Cap (Falk Minow Services, Germany) according to the international 10-10 system [39] with additional non-standard electrodes (AF1, AF2, PO1, PO3) at frontal and occipital sites spaced equally between the standard electrodes. Cz served as a reference electrode and a ground electrode was placed at the forehead. Impedances of all electrodes were kept below 5 kΩ. Vertical and horizontal electrooculograms (VEOG and HEOG) were measured for off-line correction of eye movements and blink artifacts. All signals were continuously recorded in AC-mode and sampled at 500 Hz (gain = 1000, low pass filter = 70 Hz, high pass filter = 0.05 Hz) using Synamps and NeuroScan software [40].

The EEG raw data were filtered (low pass = 30 Hz, 24 dB/oct, high pass = 0.1 Hz, 24 dB/oct, 50 Hz notch), segmented (200 ms pre- to 1300 ms poststimulus), corrected for blinks and eye movements [41], and screened for artifacts using Brain Vision Analyzer 1.05 [42]. Trials containing artifacts were rejected (minimal/maximal amplitude ± 150 μV, maximum voltage difference between neighboring sampling points 50 μV, maximal allowed absolute difference of two values in a segment 150 μV). Artifact-free EEG epochs were averaged for each subject, condition, and electrode. All epochs were aligned to the 200-ms prestimulus baseline and were rereferenced to an average reference.

### Statistical analysis

One subject (male control) was excluded from the analysis of classifications, reaction times, and ERP data because his task behavior indicated that he misunderstood the task. Five participants (1 social phobic, 2 spider aficionados, 2 controls) were excluded from ERP analysis: one because of extreme theta/alpha activity, three because of no detectable component structure, one because of problems with blink artifact correction.

For data analysis, linear mixed effects models [43] were implemented in all analyses of variance (ANOVAs) using SAS 9.1 [44]. Subjects served as a random and Picture as a repeated effect, whereas all other factors were fixed effects. Significant effects in an ANOVA were further analyzed by calculating contrasts, where rejection of the null hypothesis was controlled by Holm's sequential rejection algorithm [45]. Original *p*-values that remained significant after α-correction are reported below.

### Analysis of valence and arousal ratings

Valence and arousal ratings were analyzed by an ANOVA with between factors Anxiety (high for social and spider phobic participants, low for controls and spider aficionados), Relevance (high for spider phobic individuals and spider aficionados, low for controls and social phobic participants), and repeated measures factor Picture (Pictures 1 to 7).

### Analysis of classification frequencies

For the analysis of stimulus classifications all wrong responses (i.e., when subjects indicated that they made an incorrect response) were excluded from further analysis. For each picture in each series the relative response frequencies for each category (spider, flower, neither/nor) were calculated (i.e., the number of classifications of this picture as a spider, a flower or neither/nor, divided by the total number of correct answers to this picture by the subject). Then, the relative frequencies of the corresponding pictures in each series (e.g. the three flower anchors) were averaged over the three different flower/spider series.

A mixed Rasch model was used to analyze the response tendencies of each group. The probability of a subject to classify a stimulus as a spider was modeled as exp(z)/(1+exp(z)), where z = α_So_x_So_+ α_SP_x_SP _+ α_SA_x_SA _+ Σ_i _δ_i_x_i _+ ε. In this formula, α denotes the groups' latent traits with the control group's trait set to 0, δ_i _the i^th ^stimulus item difficulty, ε a normally distributed error term with mean 0 and all x indicator variables. High values of α and δ thus imply high probabilities of classification as a spider. Model parameters were fitted with the nonlinear mixed models procedure of SAS 9.1 [SAS Institute, Inc, see [46]].

### Analysis of response latencies

Reaction times were analyzed in two ways: first, depending on the stimulus type presented (stimulus-dependent analysis) and second, depending on the subject's response (classification of a stimulus as spider, flower or neither/nor: response-dependent analysis). All trials were excluded in which subjects gave no response, indicated that the answer was wrong, or the reaction time was below 150 ms or more than 2 *SD *from the individual mean over the stimuli or responses of the same type. In this way, 5.0% of trials were excluded from the response-locked and 5.2% from the stimulus-locked analysis. Mean reaction times in response to each picture were calculated. Then, the reaction times of the corresponding pictures in the three series were averaged.

In the stimulus-dependent analysis of RT, an ANOVA with between factors Anxiety and Relevance and repeated measures factors Picture was calculated. In the response-dependent analysis of RT, an ANOVA with between factors Anxiety and Relevance and repeated measures factor Classification (spider, flower, neither/nor) was calculated. Valence and arousal ratings were included in the ANOVA as covariates wherever preconditions for ANCOVA were met. However, no significant influence was found. Therefore, covariates were excluded from the final analysis. A similar analysis for response dependent data would not make sense.

### Analysis of ERPs

P1 and P2 peak amplitudes were detected on electrodes O1, Oz, and O2 in the time intervals [50 ms, 130 ms] and [190 ms, 270 ms], respectively. N170 peak amplitudes were detected on electrodes P7 and P8 in the time interval [190 ms, 220 ms]. Parietal mean amplitudes were exported in the time intervals [300 ms, 380 ms] for P3 and [380 ms, 580 ms] for P4 at Pz, where the late parietal positivity showed its peak amplitude. All ERPs were analyzed stimulus- as well as response-dependent as detailed above.

In the stimulus-dependent analysis, P1 and P2 amplitudes as well as mean amplitudes were analyzed by an ANOVA with between factors Anxiety and Relevance and repeated measures factors Picture and Laterality (left, central, right). N170 amplitudes were analyzed by an ANOVA with between factors Anxiety and Relevance and repeated measure factors Picture and Laterality (left, right). In the response-dependent analysis, the factor Response Category (spider, flower, neither/nor) takes the place of the factor Picture in the stimulus-dependent analysis. Valence and arousal ratings as well as response latencies were included as covariates in the stimulus dependent analysis of P1, N170, P3, and P4 amplitudes, and similarly, response dependent reaction times were included as covariates in the response dependent analysis of P1, N170, P3, and P4 amplitudes. Because no significant influence was observed, the covariates were excluded in the final analyses.

## Results

### Valence and arousal ratings

**Figure 2:**
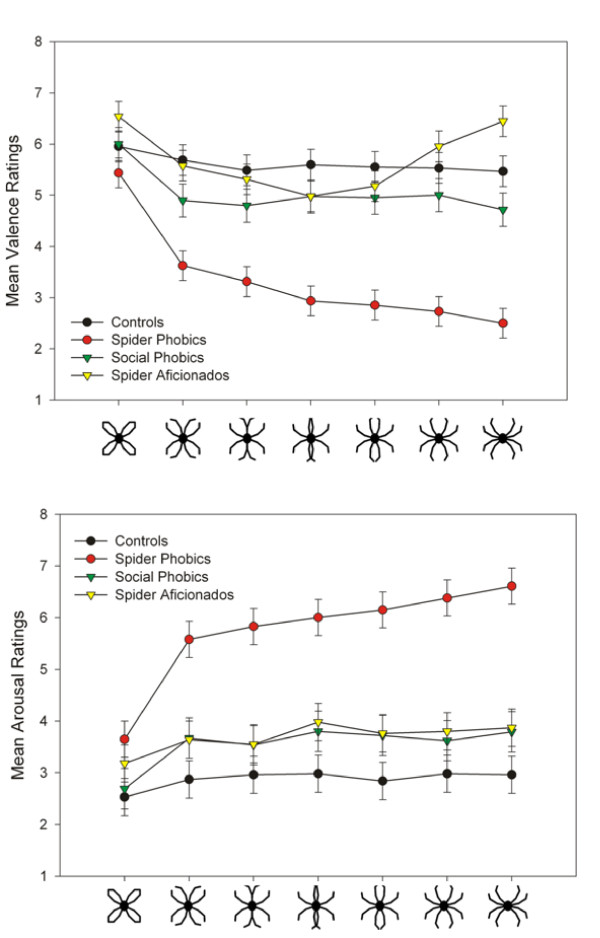
**Valence and arousal ratings**. Mean valence (upper row) and arousal ratings (lower row) and standard errors for the series of schematic spider/flower pictures for each group. *Note*. The SAM scale (Lang, 1980; Bradley & Lang, 1994) ranged from 1 to 9 with 1 = highly unpleasant/low arousing and 9 = highly pleasant/highly arousing.

Valence ratings revealed main effects of Anxiety, *F*(1,55) = 32.42, *p *< .0001, Relevance, *F*(1,55) = 9.73, *p *= .003, and Picture, *F*(6,330) = 27.13, *p *< .0001. Furthermore, the interactions Anxiety × Picture, *F*(6,330) = 7.94, *p *< .0001, Relevance × Picture, *F*(6,330) = 5.23, *p *< .0001, and Anxiety × Relevance, *F*(1,55) = 12.27, *p *= .0009, as well as the three-way interaction Anxiety × Relevance × Picture, *F*(6,330) = 2.80, *p *= .01, were significant. Whereas groups did not differ in valence ratings for picture 1, a specific contrast revealed that spider phobic individuals rated pictures 2–7 as more unpleasant than the other groups, *t*(55) = -3.70, *p *= .0005, while spider aficionados rated pictures 6 and 7 as more pleasant than controls and social phobic individuals, *t*(40) = 2.70, *p *= .01 (compare Figure 2).

Arousal ratings revealed main effects of Anxiety, *F*(1,55) = 16.79, *p *= .0001, Relevance, *F*(1,55) = 20.31, *p *< .0001, and Picture, *F*(6,330) = 23.72, *p *< .0001. Furthermore, interactions of Anxiety × Picture, *F*(6,330) = 7.49, *p *< .0001, Relevance × Picture *F*(6,330) = 3.17, *p *= .005, and Anxiety × Relevance, *F*(1,55) = 4.32, *p *= .04, as well as the three-way interaction Anxiety × Relevance × Picture, *F*(6,330) = 2.37, *p *= .03, were significant. Whereas groups did not differ in arousal ratings for picture 1, a specific contrast revealed that spider phobic individuals rated pictures 2–7 as more arousing than the other groups, *t*(55) = 2.23, *p *= .03 (compare Figure 2).

### Classifications

**Figure 3:**
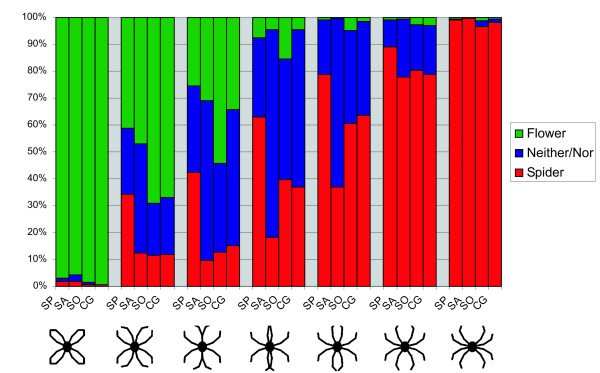
**Classifications**. Classifications: Probability of different groups (SP = Spider Phobics, SA = Spider Aficionados, SO = Social Phobics, CG = Control Group) to identify pictures as flower, neither/nor, or spider.

Figure 3 shows the probabilities of each group to identify each stimulus as a spider, a flower or neither/nor. Spider phobic individuals were more likely to classify a stimulus as a spider, α_SP _= 1.83, *t*(57) = 2.37, *p *= .02, while social phobic participants and spider aficionados did not differ from controls, all *p *> .39. The first three pictures in the series were likely *not *to be classified as spiders, picture 1 δ_1 _= -7.61, *t*(57) = -12.36, *p *< .0001, picture 2 δ_2 _= -2.97, *t*(57) = -5.24, *p *< .0001, picture 3 δ_3 _= -2.59, *t*(57) = -4.57, *p *< .0001. The last two pictures in the series were likely to be classified as spiders, picture 6 δ_6 _= 2.18, *t*(57) = 3.87, *p *= .0003, picture 7 δ_7 _= 5.09, *t*(57) = 8.68, *p *< .0001.

Separate Rasch models by group were calculated to identify item difficulties, i.e., the propensity of classifying a stimulus as a spider, for each group. Results are shown in Table 2.

**Table 2:** Item difficulties δ for each stimulus by group

							
Spider Phobics	-6.77***	-1.48	-.58	1.42	3.03*	4.15***	7.08***
Spider Aficionados	-6.93***	-2.79***	-3.38***	-1.91*	-.42	1.82*	6.09***
Social Phobics	-7.40***	-3.03***	-2.82**	-.39	.81	2.02*	4.16***
Controls	-6.32***	-2.93***	-2.51***	-.67	.91	1.87**	4.83***

Negative δ-values show a propensity to be classified as a flower; positive δ-values show a propensity to be classified as a spider. Significances: * *p *< .01, ** *p *< .001, *** *p *< .0001

### Reaction times

#### Stimulus dependent analysis of RTs

**Figure 4:**
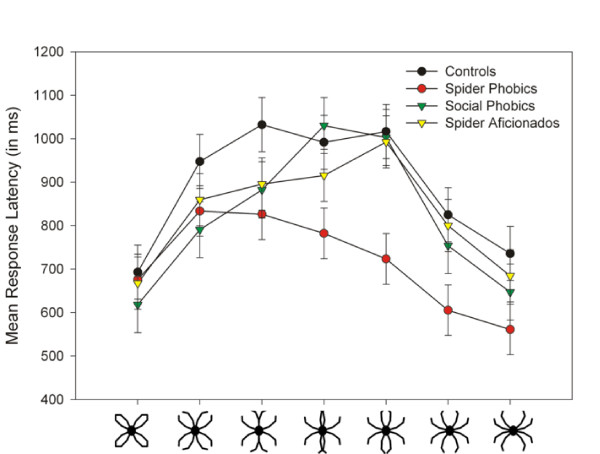
**Stimulus dependent reaction times**. Stimulus dependent RTs: Mean response latency (in ms) and standard errors for the classification of each picture depicted separately for each group.

ANOVA revealed a main effect of Picture, *F*(6,324) = 59.04, *p *< .0001, as well as interactions of Anxiety × Picture, *F*(6,324) = 2.86, *p *= .01, Relevance × Picture, *F*(6,324) = 2.97, *p *= .008, and Anxiety × Relevance × Picture, *F*(6,324) = 4.33, *p *= .0003. As can be seen in Figure 4, the more unequivocal pictures 1 and 7 were identified faster than the more equivocal pictures 3, 4, and 5. Therefore an ANCOVA with a numerical variable Picture to second order was calculated that revealed a main effect of Picture, *F*(1,340) = 187.13, *p *< .0001, as well as interactions Relevance × Anxiety, *F*(1,54) = 4.68, *p *= .04, Picture × Relevance, *F*(1,340) = 11.94, *p *= .0006, and Picture × Relevance × Anxiety, *F*(1,340) = 6.45, *p *= .01. Furthermore, quadratic terms of Picture were found in interactions Picture × Picture, *F*(1,340) = 208.62, *p *< .0001, Picture × Picture × Relevance, *F*(1,340) = 9.53, *p *= .002, and Picture × Picture × Relevance × Anxiety, *F*(1,340) = 3.94, *p *= .05. The specific contrast testing group differences for the more "spider-like" pictures 3 to 7, including the quadratic Picture term, revealed a difference between spider phobic individuals and all other groups, *t*(54) = 2.99, *p *= .004, indicating that spider phobic individuals identified these stimuli faster than the control groups.

#### Response dependent analysis of RTs

The ANOVA revealed a main effect of Response Category, *F*(2,98) = 42.71, *p *< .0001. A subsequent contrast indicated that the response neither/nor took longer than the responses spider or flower, *t*(98) = 9.24, *p *< .0001. Furthermore, interactions of Anxiety × Response Category, *F*(2,98) = 4.28, *p *= .02, Relevance × Response Category, *F*(2,98) = 3.47, *p *= .04, and Relevance × Anxiety × Response Category, *F*(2,98) = 4.98, *p *= .009, were significant. Subsequent analyses by group showed that spider phobic participants gave the answer spider faster than the answer flower, *F*(1,15) = 19.15, *p *= .0005, while there was no effect of Response Category for the other groups, all *p *> .17.

### Analysis of P1

#### Stimulus dependent analysis

**Figure 5:**
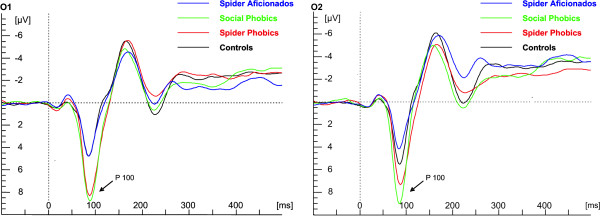
**Stimulus dependent analysis of early ERPs**. Stimulus dependent analysis of ERPs on electrode O1 (left) and O2 (right) for each group.

A main effect of Anxiety, *F*(1,49) = 9.42, *p *= .004, showed that (spider and social) phobic individuals exhibited generally larger P1 amplitudes than controls and spider aficionados (Figure 5). The main effect of Picture, *F*(6,294) = 4.39, *p *= .0003, revealed that more ambiguous pictures (3 and 4) led to larger P1 amplitudes than more unequivocal pictures (1, 2, 5, 6, 7), *t*(294) = 4.84, *p *< .0001. Testing for a quadratic effect of Picture as a continuous covariate revealed a significant effect, *F*(1,1053) = 20.35, *p *< .0001, with a negative coefficient (β = -.07) indicating a parabola that opens downwards and confirming the above effect. The ANOVA revealed a main effect of Laterality, *F*(2,104) = 4.79, *p *= .01, indicating that larger P1 amplitudes were observed at lateral, as compared to central electrodes, *t*(104) = 2.99, *p *= .003.

#### Response dependent analysis

**Figure 6:**
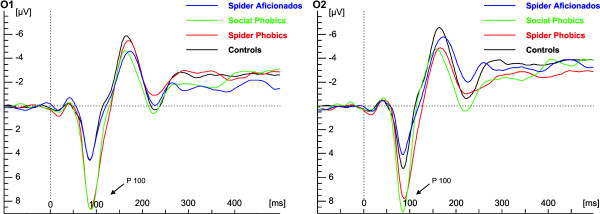
**Response dependent analysis of early ERPs**. Response dependent analysis of ERPs on electrode O1 (left) and O2 (right) for each group.

The ANOVA revealed main effects of Response Category, *F*(2,89) = 4.00, *p *= .02, Anxiety, *F*(1,49) = 9.42, *p *= .004, and Laterality, *F*(2,104) = 4.77, *p *= .01. Phobic individuals showed larger P1 amplitudes than controls and spider aficionados (Figure 6). If subjects categorized a stimulus as neither/nor, P1 amplitudes were larger compared to when they categorized the stimulus as flower or spider, *t*(89) = -2.75, *p *= .007, with no difference between flower and spider categorizations, *t*(89) = .65, *p *= .52. Finally, P1 amplitudes were larger at lateral as compared to central electrodes, *t*(104) = 3.01, *p *= .003.

### Analysis of N170

#### Stimulus dependent analysis

The ANOVA revealed main effects of Picture, *F*(6,294) = 4.87, *p *< .0001, and Laterality, *F*(1,49) = 8.38, *p *= .006, as well as a trend for an interaction of Picture × Laterality, *F*(6,294) = 1.99, *p *= .07. Larger N170 amplitudes for the more unequivocal pictures 1, 2, 5, 6, and 7 were observed compared to the more ambiguous pictures 3 and 4, *t*(294) = 3.60, *p *= .0004. Furthermore, N170 amplitudes were larger at right compared to left hemispheric electrode sites, *F*(1,49) = 8.38, *p *= .006.

#### Response dependent analysis

The ANOVA revealed main effects of Response Category, *F*(2,89) = 5.27, *p *= .007, and Laterality, *F*(1,49) = 6.70, *p *= .01, indicating that N170 amplitudes were generally larger when subjects classified the stimulus as a flower than if they classified it as a spider or as neither/nor, *t*(89) = -3.23, *p *= .002, and that amplitudes were larger over the right hemisphere.

### Analysis of P3

#### Stimulus dependent analysis

**Figure 7:**
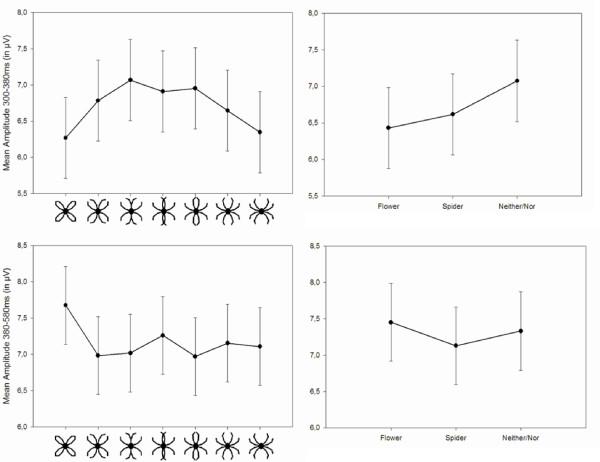
**Stimulus and response dependent analysis of late ERPs**. Mean amplitudes in the P3 (upper row) and P4 (lower row) latency range for stimulus (left) and response dependent analysis (right).

The ANOVA showed a main effect of Picture, *F*(6,294) = 3.87, *p *= .001 (Figure 7). Smallest amplitudes were observed for the more unequivocal anchor stimuli, whereas largest amplitudes were observed for the more ambiguous pictures (3 to 4). Testing for a quadratic effect of Picture as a continuous covariate revealed a significant effect, *F*(1,313) = 21.28, *p *< .0001, with a negative coefficient (β = -.08) indicating a parabola that opens downwards.

#### Response dependent analysis

A main effect of Response Category, *F*(2,89) = 5.30, *p *=.007, was observed (Figure 7). Larger positivities resulted when participants classified a stimulus as neither/nor rather than as a spider or flower, which was confirmed by a subsequent contrast, *p *= .003.

### Analysis of P4

#### Stimulus dependent analysis

A main effect of Picture, *F*(6,294) = 2.12, *p *= .05, was observed (Figure 7). Largest positivities resulted for picture 1 (the flower anchor picture) compared to all other pictures, which was confirmed by a subsequent specific contrast, *p *=.001.

#### Response dependent analysis

No significant main effects or interactions were observed.

## Discussion

This study found behavioral evidence for an interpretative bias in spider phobia: spider phobic individuals showed an enhanced latent trait to classify a stimulus as a spider compared to the control groups composed of non-fearful individuals, social phobics and spider aficionados. Furthermore, while the response latencies of the control groups were a function of ambiguousness of stimuli following a quadratic relationship, spider phobic individuals showed a faster classification of stimuli resembling spiders, starting with picture 3 in the series. Finally, spider phobic individuals rated ambiguous stimuli in the series – starting with the first opening of the petals of the flower – as more arousing and unpleasant than the control groups.

Whereas results on a behavioral level confirmed the presence of an interpretative bias in spider phobics, analysis of ERP components did not reveal cortical correlates of such a bias. As expected, P1 amplitude was generally larger in phobics (social and spider phobic individuals) as compared to controls, possibly indicating an enhanced vigilance in phobia. Furthermore, P1 amplitude was influenced by the ambiguousness of stimuli: in the stimulus-dependent analysis more ambiguous pictures led to larger P1 amplitudes than more unequivocal pictures; correspondingly, larger P1 amplitudes were observed for neither/nor classifications than for flower or spider classifications in the response-dependent analysis. N170 amplitude did not differentiate spider phobics from spider-non-phobics and was only modulated by the presented stimulus or response category, respectively. P3 amplitudes also showed no specific effect for the spider phobic group, but the amount of positive deflection was modulated by the ambiguousness of pictures, following a quadratic parabola with largest positive deflections for the most ambiguous and smallest for the unequivocal anchor pictures in the stimulus dependent analysis. Correspondingly, in the response dependent analysis largest positivities were observed for neither/nor compared to spider or flower classifications. Finally, P4 amplitudes were also not indicative of spider phobia specific processes, but showed a general influence of Gestalt properties: presumably due to an oddball effect, the only closed Gestalt (the flower anchor), elicited largest amplitudes in this latency range. This effect was also visible as a trend in ERPs of the response dependent analysis, but did not reach significance here.

Effects of Anxiety and/or Relevance were only found in behavioral measures and in the P1. As detailed above, in the P1, the main effect of Anxiety revealed a phobia-specifically increased amplitude. In each of the behavioral measures, there was an interaction of Anxiety × Relevance, showing that behavioral differences are not due solely to phobia or to subjective (positive or negative) relevance of spiders, but are specific to spider phobia as such.

### Interpretative bias in spider phobia and its implications

The analysis of classification frequencies confirmed that stimuli were rated more often as "spider-like" or "flower-like" the closer their position in the flower/spider series to the corresponding anchor picture. Pictures in mid-positions were largely classified as neither/nor. A clear threshold in the series beyond which stimuli were perceived as spiders was not obvious. Classifications changed in a rather continuous manner.

Spider phobics exhibited a stronger latent trait to classify a picture as a spider than controls as shown by the Rasch model, suggesting either an interpretive bias or a stimulus generalization effect. Separate Rasch models by group revealed that picture 1 was likely to be identified as a flower by all groups, but pictures 2 and 3 were only classified as a flower by non-spider phobic participants. Picture 4 was classified by spider aficionados as a flower, and picture 5 was already classified as a spider by spider phobic individuals. Pictures 6 and 7 were classified as spiders by all groups. Thus, while spider phobic individuals were liberal in classifying ambiguous stimuli as spiders, starting at picture 5 and even 4 if using a one-sided test, spider aficionados were more conservative in labeling ambiguous stimuli such as picture 4 as a flower. In all groups, the progression from stimuli perceived as flower-like to those perceived as spider-like was smooth and not abrupt.

These results are in agreement with a recent study by Becker & Rink [6] using a signal detection paradigm in which participants were asked to decide whether a picture of either a spider, beetle, or butterfly or rather a neutral stimulus was presented. Spider phobics were more liberal in assuming that they had seen a spider or a beetle, suggesting a *cognitive *interpretive bias. Becker & Rink argued that their results suggest that spider phobic persons might show a generalized bias to interpret rather harmless stimuli as threatening ones. A similar bias has also been observed in individuals with social phobia, who interpret (ambiguous) social situations as more threatening and draw more negative inferences from the available social stimuli than controls [3,5].

The results of the present study are consistent with a stimulus generalization effect, i.e. that a given response can be elicited to some degree by a range of similar stimuli. That the bias might be not merely cognitive is also suggested by the faster responses to ambiguous stimuli in spider phobic persons. The observed pattern is reminiscent of the mechanisms in PTSD, where stimulus generalization also accounts for the exacerbation and extension of symptoms to additional stimuli and as a consequence, stimuli only peripherally related to the trauma can trigger intrusions and flashbacks. Whether the present findings are due to a stimulus generalization effect or a more cognitive interpretive bias thus remains open.

### Hypervigilance in phobia

According to Beck et al. [[47], p. 31], "The [anxious] patient is hypervigilant, constantly scanning the environment for signs of impending disaster or personal harm." According to Eysenck [48-50], there are two ways in which individuals high in trait anxiety show hypervigilance: *general *hypervigilance or distractability is demonstrated by a propensity to attend to any task-irrelevant stimuli presented, and *specific *hypervigilance is demonstrated by a tendency to attend selectively to threat-related rather than neutral stimuli.

In the present study, spider phobic individuals responded generally faster to spiderlike stimuli (stimulus dependent analysis) and to stimuli they classified as a spiders (response dependent analysis), as would be expected in specific hypervigilance. This corresponds to faster identification of non-schematic spiders than flowers or birds by spider phobics and faster identification of non-schematic spiders by spider phobics than by social phobics and controls [13]. On the other hand, Kolassa et al. [14] found unspecifically faster responses by spider phobics to colored schematic flowers *and *spiders. However, both the present study and Kolassa et al. [13] used more than two stimulus types and response categories, while Kolassa et al. [14] used only two stimulus types and two response categories, which renders comparisons difficult. Kolassa et al. [14] note that standard deviations in their study were quite small, which may have led to small effects reaching significance.

### Phobia-specific responses in early ERPs

The larger early visual P1 amplitudes in phobic compared to non-phobic subjects replicate earlier studies that also observed enhanced P1 amplitudes in phobic compared to non-phobic subjects when processing schematic flower/spider stimuli or schematic emotional faces [14,51,52]. In the light of the hypervigilance model detailed above, the enhanced P1 amplitude may be a correlate of an increased readiness of phobic individuals' cortex to react to new visual stimuli. Thus, the P1 amplitude differential could be interpreted as indicative of a cortical correlate of general hypervigilance in the anxiety disorder spectrum.

### LPPs

The results of LPPs (P3 and P4) fit very well with Johnson's [53] triarchic model of P3 amplitude, which posits that *information transmission, subjective probability *and *stimulus meaning *strongly influence LPP amplitudes. Regarding information transmission, Johnson's model assumes that unequivocal pictures lead to larger amplitudes than more ambiguous pictures, as was found in the present results on P3. Next, subjectively less probable stimuli should also lead to higher amplitudes, which again fits the present results from the response-dependent analysis: the least common reply, neither/nor, was associated with a higher P3 than the other two response categories.

P4 amplitude was higher for the flower anchor than for the other pictures, which may be discussed by extending Johnson's model to the P4. As detailed above, subjectively less probable stimuli should lead to higher amplitudes. In the present paradigm, only the flower anchor had a closed Gestalt, while all other pictures had an open Gestalt. Thus, the least common Gestalt may have been associated with a lower subjective probability.

It thus appears possible that the influence of subjective probability was larger than the influence of stimulus meaning in terms of valence or arousal, explaining why the present study did not find enhanced LPPs in spider phobic individuals in response to schematic spiders, as was observed in previous studies with schematic flower/spider stimuli [14] or with photographic spider pictures [13].

### The N170

More unequivocal pictures led to larger N170 amplitudes than more equivocal ones, in an interesting parallel to the P3 findings. However, in spite of ongoing controversy on the role of the N170 [54-56], there appears to be no account of an influence of ambiguity on N170, thus the meaning of this finding remains unclear.

In addition, N170 amplitudes were higher when participants classified stimuli as flowers than when they classified them as spiders or neither/nor, in contrast to Kolassa et al. [14], who found that schematic spiders elicited higher N170 amplitudes than schematic flowers. However, comparing the two paradigms is not easy: in our 2006 study, only two stimulus categories were shown, not an entire spectrum as in the present case, and response categories were spider vs. flower, not spider vs. flower vs. neither/nor. Importantly, the present result derives from participants' subjective classification of stimuli, not from objective stimulus types.

### Gestalt properties

Item difficulties as revealed by the Rasch model allow us to group pictures 1–3 as easily identifiable as flowers (δ < 0), pictures 4 and 5 as indeterminate (δ not significantly different from zero), and pictures 6 and 7 as identifiable as spiders (δ > 0). ERPs (P1, N170 and P3) consistently show a difference between pictures 3 and 4, on the one hand, and pictures 1, 2, 5–7, on the other hand. What differentiates these groups of pictures?

A first explanation for the differences between these groups of stimuli can be found in the Gestalt law of closure [57]: open shapes make one perceive a visual pattern as incomplete, and our minds tell us to close small gaps and complete unfinished forms. This is possible for pictures 1 to 3, but starting with picture 4, the gaps between the lines are so pronounced that the law of closure may no longer apply and the connecting of lines to form petals is no longer possible. Instead, participants may have connected the top two middle lines and the bottom two middle lines in pictures 4 and 5, forming an indeterminate non-spider-like stimulus. Finally, pictures 6 and 7 were so clearly spider-like that the law of closure may no longer have been able to change this classification.

## Conclusion

Behavioral evidence for an interpretive bias in spider phobia was observed, extending findings on the existence of a negative interpretation bias in social phobia. Clinical experience points to similar biases in other anxiety disorders such as PTSD: stimuli only peripherally related to the trauma can trigger intrusions and flashbacks in PTSD patients. In light of the present study, this finding may be explainable as a consequence of stimulus generalization in anxiety disorders. Whether the effect observed in spider phobic individuals is due to a more cognitive interpretive effect or due to stimulus generalization remains subject to future studies.

In agreement with earlier studies, social and spider phobic persons exhibited generally enhanced visual P1 amplitudes indicative of hypervigilance in phobia and possibly of the preattentive feature detectors as postulated by Öhman [20]. Ambiguity-related modulations of N170 and P3 as well as Gestalt-related modulations of P4 were found, in accordance with Johnson's [53] triarchic model of LPP amplitude.

## Competing interests

The author(s) declare that they have no competing interests.

## Authors' contributions

ITK designed the study, recruited and tested subjects, performed the analyses and wrote the manuscript. AB recruited subjects, acquired data and supported the analysis. RL recruited and tested spider aficionados. SK programmed the paradigm and supported statistical analysis and write-up of the manuscript. IP designed the Rasch models. WHRM designed the study. FM designed the study and created the stimuli.
